# Biomechanical properties of articular cartilage in different regions and sites of the knee joint: acquisition of osteochondral allografts

**DOI:** 10.1007/s10561-024-10126-3

**Published:** 2024-02-06

**Authors:** Yongsheng Ma, Qitai Lin, Xueding Wang, Yang Liu, Xiangyang Yu, Zhiyuan Ren, Yuanyu Zhang, Li Guo, Xiaogang Wu, Xiangyu Zhang, Pengcui Li, Wangping Duan, Xiaochun Wei

**Affiliations:** 1 Department of Orthopaedics, Second Hospital of Shanxi Medical University, No. 382, Wuyi Road, Taiyuan, 030001 Shanxi China; 2 Shanxi Key Laboratory of Bone and Soft Tissue Injury Repair, Taiyuan, 030001 China; 3 Institute of Biomedical Engineering, College of Biomedical Engineering, Taiyuan University of Technology, Taiyuan, 030024 China

**Keywords:** Articular cartilage, Biomechanics, Osteochondral allograft, Different regions, Cartilage thickness

## Abstract

Osteochondral allograft (OCA) transplantation involves grafting of natural hyaline cartilage and supporting subchondral bone into the cartilage defect area to restore its biomechanical and tissue structure. However, differences in biomechanical properties and donor-host matching may impair the integration of articular cartilage (AC). This study analyzed the biomechanical properties of the AC in different regions of different sites of the knee joint and provided a novel approach to OCA transplantation. Intact stifle joints from skeletally mature pigs were collected from a local abattoir less than 8 h after slaughter. OCAs were collected from different regions of the joints. The patella and the tibial plateau were divided into medial and lateral regions, while the trochlea and femoral condyle were divided into six regions. The OCAs were analyzed and compared for Young’s modulus, the compressive modulus, and cartilage thickness. Young’s modulus, cartilage thickness, and compressive modulus of OCA were significantly different in different regions of the joints. A negative correlation was observed between Young's modulus and the proportion of the subchondral bone (r =  − 0.4241, *P* < 0.0001). Cartilage thickness was positively correlated with Young’s modulus (r = 0.4473, *P* < 0.0001) and the compressive modulus (r = 0.3678, *P* < 0.0001). During OCA transplantation, OCAs should be transplanted in the same regions, or at the closest possible regions to maintain consistency of the biomechanical properties and cartilage thickness of the donor and recipient, to ensure smooth integration with the surrounding tissue. A 7 mm depth achieved a higher Young's modulus, and may represent the ideal length.

## Introduction

Articular cartilage (AC) has a unique ability to adapt to pressure changes by providing a surface for reducing friction and wear resistance for joints, and is thus necessary for natural articular activity. (Li et al. 2021a) However, spontaneous healing of AC lesions is rare because of a lack of innervation and poor vascular supply. (Li et al. 2021b, Mieloch et al. 2019, Risch et al. 2021) Osteochondral allograft (OCA) transplantation is used specifically for the treatment of chondral and osteochondral diseases, such as focal degenerative osteochondritis dissecans, acute traumatic avascular necrosis, or osteoarthritis primarily in the knee. (Cook et al. 2016) OCA transplantation is a biological technique that can anatomically and functionally restore damaged hyaline cartilage. (Mickevicius et al. 2015) However, integration could be impaired and the outcome of AC repair could be affected by the different biomechanical properties of the host tissue, graft, and different cartilage regions. (Mieloch et al. 2019).

The mechanical properties of materials or tissue surfaces are primarily detected by nanoindentation. (Guo et al. 2022) This study measured the micro-elasticity of the surface of porcine cartilage using a novel nanoindentation device. (Boi, et al. 2019, Guo et al. 2022, Wang et al. 2014, Yuh et al. 2021) The method offers high precision and accuracy, and provides deeper insight into the nanomechanical properties of the cartilage tissue. Furthermore, anisotropic and nonlinear biomechanical properties of cartilage structure and composition are generated during compression and tension. (Guo et al. 2022, Li et al. 2021a, McCready et al. 2022) The unconfined compression test was used to determine the compressive strength of the entire cartilage, calculate the compressive modulus through stress–strain curves, and macroscopically analyze the biomechanical properties of the AC. (Cooper et al. 2017, Nebelung et al. 2017, Shi et al. 2020, Sidharthan et al. 2021, Weitkamp et al. 2021).

The study measured the Young’s modulus of the AC surface and the compressive modulus using nanoindentation measurements and unconfined compression tests, which provided microscopic and macroscopic analysis of the biomechanical properties of AC. The primary objective of this study was to define distinct regions within the knee joint based on the biomechanical properties and thickness of the AC to provide innovative approaches for the acquisition, preservation, and implantation of OCA. The secondary objective was to assess the optimal length or the effect of the absence of the subchondral bone on the biomechanical properties of the cartilage surface.

## Methods

### Tissue harvest and preparation

Intact porcine stifle joints from skeletally mature 6–8-month-old animals were collected from a local abattoir within 8 h after slaughter and stored intact in a capsule. No animals were specially raised, bred, or sacrificed; only isolated tissues were used. The use of tissue samples in this study was approved by the Animal Ethics Committee of the Second Hospital of Shanxi Medical University.

**Fig. 1:**
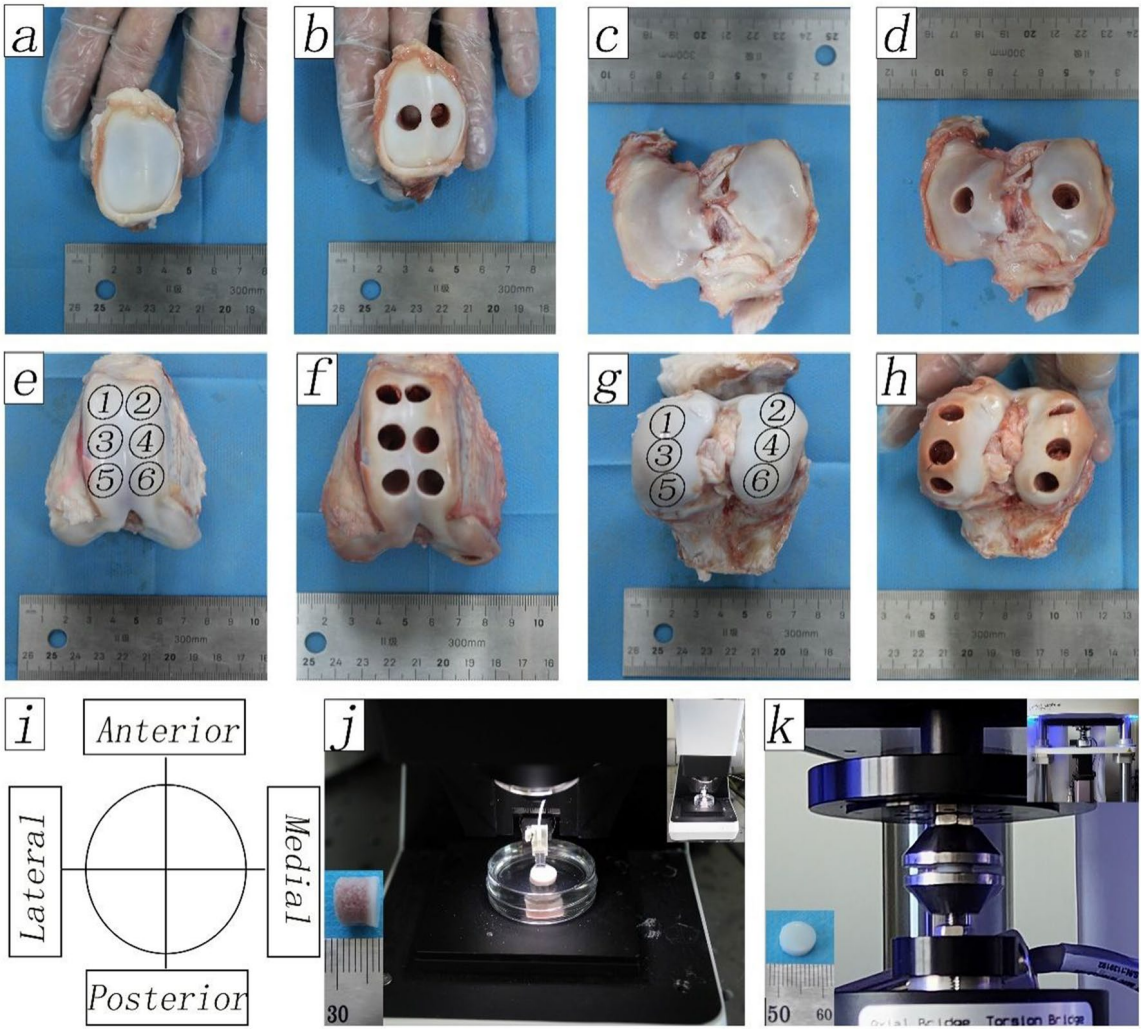
OCAs were collected from different regions and tested. **a**–**b**, The patellar division (LP and MP) and acquisition of OCAs. **c**–**d**, The tibial plateau division (LTP and MTP) and acquisition of OCAs. **e**–**f**, The trochlea division (six regions) and acquisition of OCAs. **g**–**h**, The femoral condyle (six regions) and acquisition of OCAs. **i**, Orientation diagram. **j**, Nanoindentation measurement. **k**, Uniaxially unconfined compression test. OCAs: osteochondral allografts; LP: lateral patella; MP: medial patella; LTP: lateral tibial plateau; MTP: medial tibial plateau

The stifle joints of the pigs were immersed in phosphate-buffered saline (PBS) and transferred to the operating room in refrigerated bags. The soft tissues were dissected to expose the patella. After checking the integrity of the anterior and posterior cruciate ligaments, the ligaments were severed to completely expose the femoral condyle. The joints were checked macroscopically to ensure the absence of osteoarthritic changes. A total of 22 stifle joints were used: four were used for histological staining, nine were used to measure the biomechanical properties of the cartilage surface, and nine were used to measure the compressive modulus of the cartilage. An OCA transplant instrument (Smith & Nephew Inc. USA) was used to obtain OCAs from the different regions of the stifle joints (Fig. 1). The patella and tibial plateau were divided into medial and lateral regions, and the trochlea and femoral condyle were divided into six regions (Table 1). During tissue procurement, the cartilage surface was periodically rinsed with PBS to maintain hydration and remove blood, bone, and cartilage residues from the OCAs.

**Table 1:** Regional division and OCAs number in different sites

Site	Region				OCAs number		
Patella					18		
	Lateral			LP		9	
	Medial			MP		9	
Tibial plateau					18		
	Lateral			LTP		9	
	Medial			MTP		9	
Trochlea					54		
	Lateral					27	
		AL	Region 1	ALT			9
		CL	Region 3	CLT			9
		PL	Region 5	PLT			9
	Medial					27	
		AM	Region 2	AMT			9
		CM	Region 4	CMT			9
		PM	Region 6	PMT			9
Femoral condyle					54		
	Lateral					27	
		AL	Region 1	ALFC			9
		CL	Region 3	CLFC			9
		PL	Region 5	PLFC			9
	Medial					27	
		AM	Region 2	AMFC			9
		CM	Region 4	CMFC			9
		PM	Region 6	PMFC			9

*OCAs* osteochondral allografts, *AL* anterolateral, *CL* centrolateral, *PL* posterolateral, *AM* anteromedial, *CM* centromedial, *PM* posteromedial, *LP* lateral patella, *MP* medial patella, *LTP* lateral tibial plateau, *MTP* medial tibial plateau, *ALT* anterolateral trochlea, *CLT* centrolateral trochlea, *PLT* posterolateral trochlea, *AMT* anteromedial trochlea, *CMT* centromedial trochlea, *PMT* posteromedial trochlea, *ALFC* anterolateral femoral condyle, *CLFC* centrolateral femoral condyle, *PLFC* posterolateral femoral condyle, *AMFC* anteromedial femoral condyle, *CMFC* centromedial femoral condyle, *PMFC* posteromedial femoral condyle

### Nanoindentation measurement

Osteochondral tissues were obtained from different regions of the stifle joints, with one sample taken from each region (diameter 8.5 mm and length 7 mm). The harvested tissues were immersed in lactated Ringer solution and biomechanical testing was performed in one day. The first and second rounds of measurement were conducted using 7 and 4 mm OCAs (the subchondral bone was trimmed off with a surgical scalpel to obtain 4 mm OCAs), respectively. Subsequently, the cartilage layer was obtained by excising subchondral bone from a cartilage-bone interface with a scalpel. The thickness of the AC was then measured at four different locations using a Vernier caliper and averaged.

During the nanoindentation measurement, the OCA sample was fixed inside a culture dish with cyanoacrylate superglue and submerged in PBS to maintain tissue hydration. The culture dish was securely placed on the dedicated stage of the indenter, with the superficial layer of the cartilage facing the head of the device. (Amann et al. 2017, Guo et al. 2022, Martorina et al. 2017) A probe with a stiffness of 58.24 N/m and 30 µm tip radius was used. (Guo et al. 2022) For each measurement, the same spot of the sample was used for each single indentation, and four unique points were set for each sample measured. (Zhao et al. 2021) The maximum depth of the indentation was 10.0 µm with a Poisson’s ratio of 0.5. The load-indentation determined Young’s modulus using the Hertz model. (Guo et al. 2022) Data were collected after the probe made contact with the cartilage.

### Uniaxial unconfined compression test

Cartilage samples were collected from nine different joints, as described above. The subchondral bone was excised using a surgical scalpel, and cartilage thickness was measured using the caliper, following the same method. Uniaxially unconfined compression tests were performed using the ElectroForce 3200 (Bose, Eden Prairie, MN) to determine the compressive modulus of the cartilage. The mover arm was fitted with a 25 mm metal plate and fell until it made contact with the cartilage surface. An initial force of 0.2 N was exerted, and the cartilage was then compressed axially with a 20% strain at a rate of 0.01 mm per second. This strain was maintained for 120 s. Following this, a load–displacement curve was generated, and the average compressive modulus under a 10–15% strain was calculated using the following equation (Shi et al. 2020):

$$E=\frac{\sigma (stress)}{\varepsilon (strain)}=\frac{F/S}{dL/L}$$

E = Compressive modulus

F = The loading force

S = Cross-sectional area

dL = Displacement

L = Original length

### Histological assessment

Histological evaluation was carried out using osteochondral tissues obtained from four joints. Two samples were collected from each site and then immersed in 4% paraformaldehyde for 24 h, followed by decalcification in 10% EDTA, pH = 7.4, for 4 weeks. Samples were divided in half and embedded in paraffin. Sections (5 μm) along a vertical plane were collected using a rotary microtome (RM2245, Leica, Germany) to obtain a cross sectional view of the OCA and were stained with hematoxylin and eosin (H&E). Subsequently, sections underwent conventional processing (dehydration and transparency) and imaging using a digital pathology slide scanner (Pannoramic MIDI, 3DHISTECH, Hungary). The cartilage length was measured using Case Viewer scanner software.

### Statistical analysis

Data are presented as mean ± standard deviation. Statistical analyses were performed using SPSS 25.0 (IBM, NY, USA) and illustrations were plotted with GraphPad Prism 9 (prism, CA, USA). Statistical significance between the groups was determined using an unpaired, two-tailed student’s t-test to compare two groups of data or a one-way analysis of variance (ANOVA) with Bonferroni’s post-hoc test to compare three or more groups of data. Spearman’s correlation analysis was used to determine the correlations between the thickness of the different cartilage and biomechanical parameters. *P* < 0.05 was considered statistically significant.

## Results

### Nanoindentation measurements

**Fig. 2:**
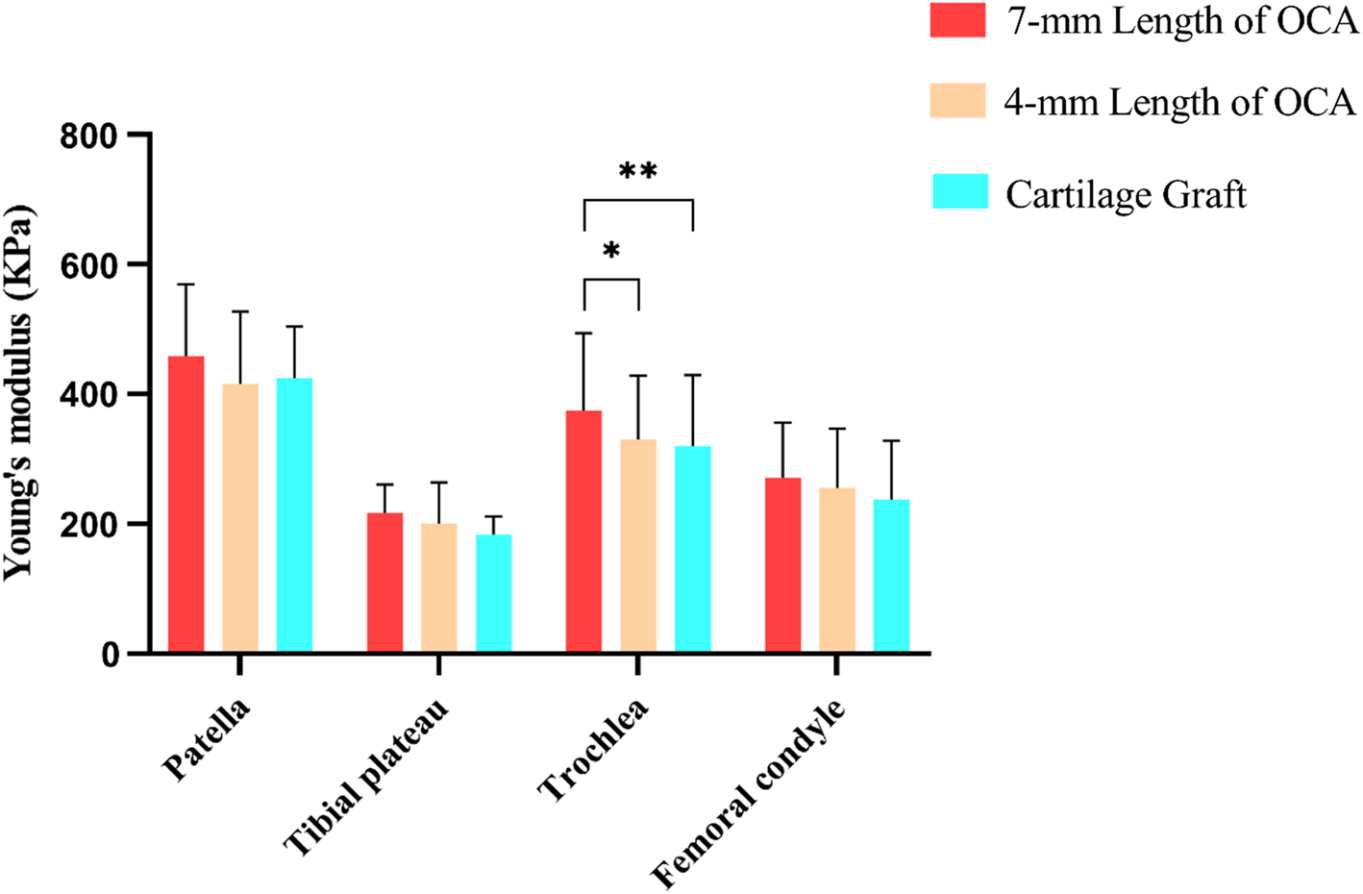
The Young's modulus of grafts of different lengths from different sites. OCA: osteochondral allograft

Measurement results of nanoindentation showed that Young's modulus of the OCAs surface of 7 mm in depth was higher than that of 4 mm OCAs and cartilage grafts, especially in the trochlea where Young's modulus of 7 mm grafts (375.26 ± 118.96 kPa) was significantly higher than that of the 4 mm grafts (330.46 ± 98.81 kPa) (*P* = 0.0294) and cartilage grafts (320.52 ± 108.91 kPa) (*P* = 0.0058) (Fig. 2).

To verify the biomechanical properties of different AC sites and different subregions, Young's modulus was compared at different sites and different subregions of 7 mm grafts, 4 mm grafts, and cartilage layer grafts. The trochlea was divided into six regions: anterolateral trochlea (ALT), centrolateral trochlea (CLT), posterolateral trochlea (PLT), anteromedial trochlea (AMT), centromedial trochlea (CMT), posteromedial trochlea (PMT). The femoral condyle was divided into six regions: anterolateral femoral condyle (ALFC), centrolateral femoral condyle (CLFC), posterolateral femoral condyle (PLFC), anteromedial femoral condyle (AMFC), centromedial femoral condyle (CMFC), posteromedial femoral condyle (PMFC) (Table 1).

Measurement of the 7-mm grafts revealed that Young's modulus of the patellar cartilage of 7-mm grafts was the highest (459.26 ± 110.30 kPa) followed by that of the trochlea (375.26 ± 118.96 kPa), femoral condyle (271.86 ± 84.70 kPa), and tibial plateau (217.03 ± 43.88 kPa) (*P* < 0.0001). In summary, the lateral and medial cartilages of different joint sites had a significant effect on biomechanics.

Young's modulus values were lower in the medial patella (MP) (453.72 ± 110.29 kPa), lateral tibial plateau (LTP) (201.49 ± 29.97 kPa), lateral femoral condyle (LFC) (253.69 ± 78.47 kPa), and lateral trochlea (LT) (312.16 ± 75.04 kPa), than in the lateral patella (LP) (464.80 ± 116.71 kPa, *P* = 0.8386), medial tibial plateau (MTP) (232.59 ± 51.47 kPa, *P* = 0.1366), medial femoral condyle (MFC) (290.02 ± 88.22 kPa, *P* = 0.1160), and medial trochlea (MT) (438.37 ± 122.25 kPa, *P* < 0.0001), respectively.

Young's modulus in the six regions of the trochlea showed the following relationships: ALT vs. AMT (*P* = 0.0046); ALT vs. CMT (*P* < 0.0001); AMT vs. PLT (*P* = 0.0318); CLT vs. CMT (*P* = 0.0394); CMT vs. PLT (*P* = 0.0003); and CMT vs. PMT (*P* = 0.0497). Other post-hoc test results were not statistically significant. The trochlear regions with the highest and lowest Young's modulus were CMT (504.59 ± 114.32 kPa) and ALT (271.09 ± 58.12 kPa), respectively.

**Fig. 3:**
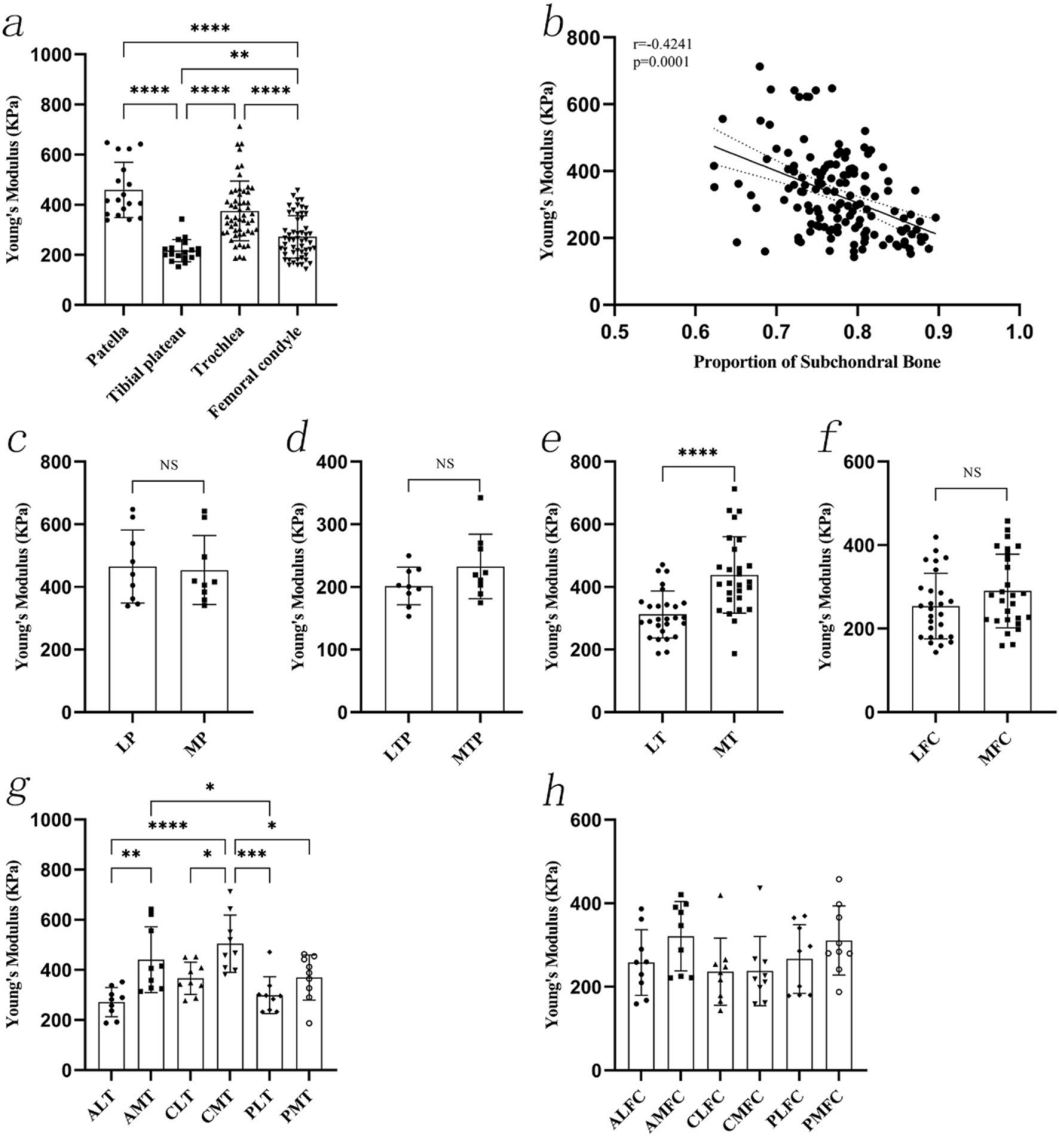
The Young's modulus of 7-mm length OCAs. **a**, The Young's modulus of OCAs from different sites. **b**, The correlation between Young's modulus of OCAs and the proportion of subchondral bone. **c**–**f**, The Young's modulus of OCAs at the LP and MP, LTP and MTP, LT and MT, LFC and MFC. **g**–**h**, The Young's modulus of OCAs at six regions of the trochlea and six regions of the femoral condyle. OCAs: osteochondral allografts; LP: lateral patella; MP: medial patella; LTP: lateral tibial plateau; MTP: medial tibial plateau. LT: lateral trochlea; MT: medial trochlea; LFC: lateral femoral condyle; MFC: medial femoral condyle; ALT: anterolateral trochlea; CLT: centrolateral trochlea; PLT: posterolateral trochlea; AMT: anteromedial trochlea; CMT: centromedial trochlea; PMT: posteromedial trochlea; ALFC: anterolateral femoral condyle; CLFC: centrolateral femoral condyle; PLFC: posterolateral femoral condyle; AMFC: anteromedial femoral condyle; CMFC: centromedial femoral condyle; PMFC: posteromedial femoral condyle

No statistically significant differences were observed between Young’s modulus between the six regions of the femoral condyle. The regions of the femoral condyle with the highest and lowest Young's modulus were AMFC (321.26 ± 82.91 kPa) and CLFC (236.28 ± 80.69 kPa), respectively. Young's modulus of cartilage was negatively associated with the proportion of the subchondral bone (r =  − 0.4241, *P* < 0.0001) (Fig. 3).

The measurement results of the 4-mm grafts showed that Young's modulus values of the patellar cartilage (416.28 ± 111.46 kPa) were the highest, followed by those of the trochlea (330.46 ± 98.81 kPa), femoral condyle (255.95 ± 91.40 kPa), and tibial plateau (200.89 ± 63.10 kPa) (*P* < 0.0001). Overall, the lateral and medial cartilages of different joint sites had a significant effect on the biomechanical properties.

Young's modulus values at the LP (402.23 ± 85.93 kPa), LTP (182.40 ± 43.87 kPa), LFC (241.25 ± 71.79 kPa) and LT (296.06 ± 95.65 kPa) were lower than those at the MP (430.33 ± 136.27 kPa, *P* = 0.6080), MTP (219.38 ± 75.95 kPa, *P *= 0.2241), MFC (270.64 ± 106.90 kPa, *P* = 0.2417), and MT (369.60 ± 104.81 kPa, *P* = 0.0027), respectively.

**Fig. 4:**
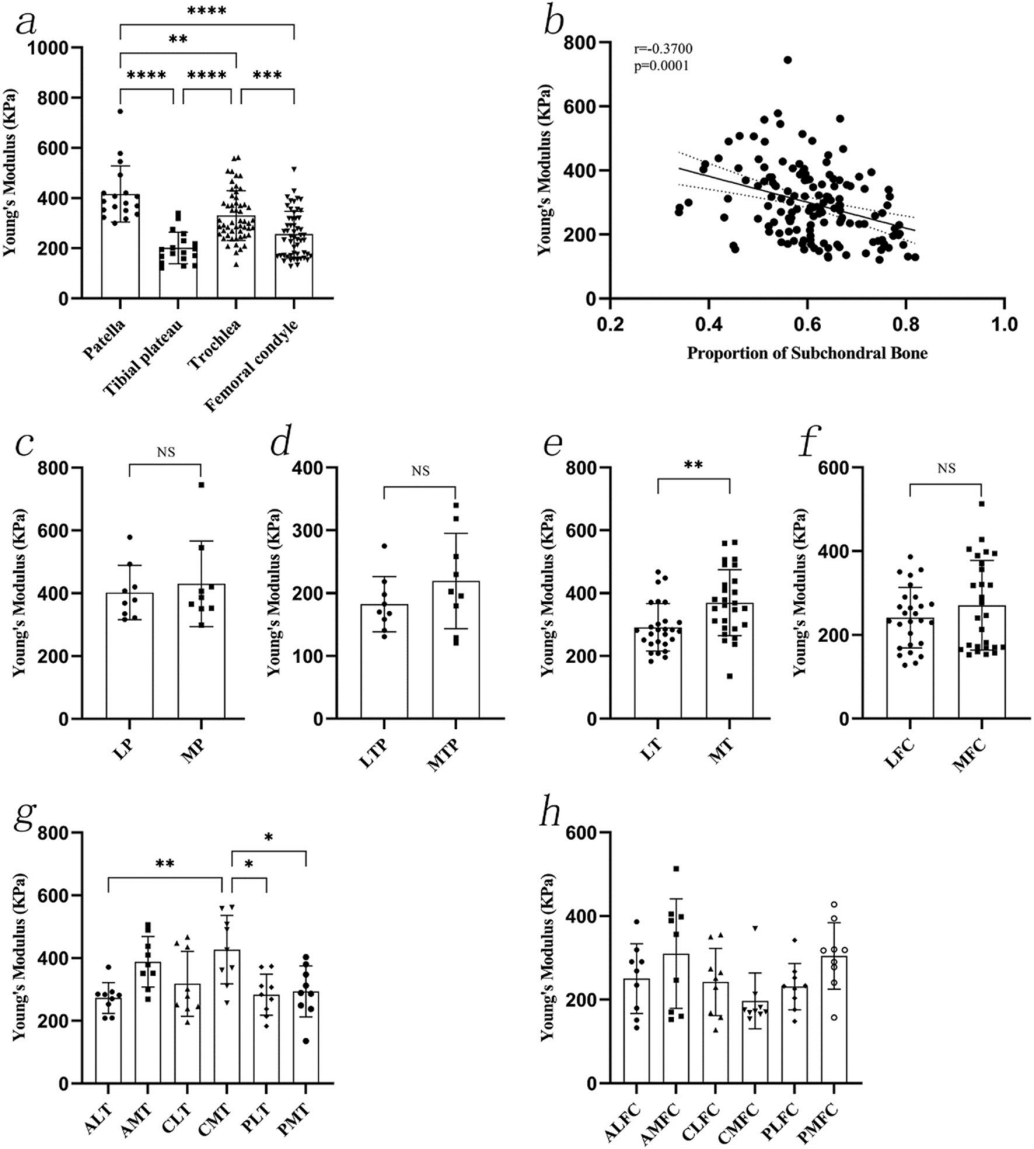
The Young's modulus of 4 mm length OCAs. **a**, The Young's modulus of OCAs from different sites. **b**, The correlation between Young's modulus of cartilage and the proportion of subchondral bone. **c**–**f**, The Young’s modulus of OCAs at the LP and MP, LTP and MTP, LT and MT, LFC and MFC. **g**–**h**, The Young's modulus of OCAs at six regions of the trochlea and six regions of the femoral condyle. OCAs: osteochondral allografts; LP: lateral patella; MP: medial patella; LTP: lateral tibial plateau; MTP: medial tibial plateau. LT: lateral trochlea; MT: medial trochlea; LFC: lateral femoral condyle; MFC: medial femoral condyle; ALT: anterolateral trochlea; CLT: centrolateral trochlea; PLT: posterolateral trochlea; AMT: anteromedial trochlea; CMT: centromedial trochlea; PMT: posteromedial trochlea; ALFC: anterolateral femoral condyle; CLFC: centrolateral femoral condyle; PLFC: posterolateral femoral condyle; AMFC: anteromedial femoral condyle; CMFC: centromedial femoral condyle; PMFC: posteromedial femoral condyle

Young's modulus in the six regions of the trochlea showed the following relationships: ALT vs. CMT (*P* = 0.0046), CMT vs. PLT (*P* = 0.0105), and CMT vs. PMT (P = 0.0225). Other post-hoc test results were not statistically significant. The region of the trochlea with the highest and lowest Young's modulus was CMT (427.04 ± 109.37 kPa) and ALT (272.67 ± 48.72 kPa), respectively. No statistically significant differences were observed between Young's modulus in the six regions of the femoral condyle. The femoral condyle region with the highest and lowest Young's modulus was AMFC (310.23 ± 130.99 kPa) and CMFC (196.83 ± 66.78 kPa), respectively. Young's modulus of cartilage was negatively correlated with the proportion of the subchondral bone (r = 0.3700, *P* = 0.0001) (Fig. 4).

Young's modulus of the patellar cartilage (424.71 ± 79.45 kPa) was the highest, followed by that of the trochlea (320.52 ± 108.91 kPa), femoral condyle (237.80 ± 90.58 kPa), and the tibial plateau (183.85 ± 28.12 kPa) (*P* < 0.0001). Overall, the lateral and medial cartilages of different joint sites had a significant effect on the biomechanical properties.

Young's modulus values at the LP (400.36 ± 65.92 kPa), MTP (175.57 ± 18.22 kPa, *P* = 0.2218), LT (296.06 ± 95.65 kPa) and LFC (208.21 ± 81.14 kPa) were lower than those at the MP (449.06 ± 87.94 kPa, *P* = 0.2025), LTP (192.13 ± 34.55 kPa), MT (344.98 ± 117.41 kPa, *P* = 0.0993), and MFC (267.38 ± 91.23 kPa, *P* = 0.0149), respectively.

No statistically significant differences were observed between Young's modulus at the six regions of the trochlea. The region of the trochlea with the highest and lowest Young's modulus were the CMT (385.44 ± 114.05 kPa) and ALT (270.65 ± 96.21 kPa), respectively. Young’s modulus in the femoral condyle regions showed a relationship between AMFC and PLFC (*P* = 0.0183). Other post-hoc test results were not statistically significant. The regions of the femoral condyle with the highest and lowest Young’s modulus were the AMFC (310.89 ± 103.77 kPa) and PLFC (175.45 ± 58.44 kPa), respectively.

**Fig. 5:**
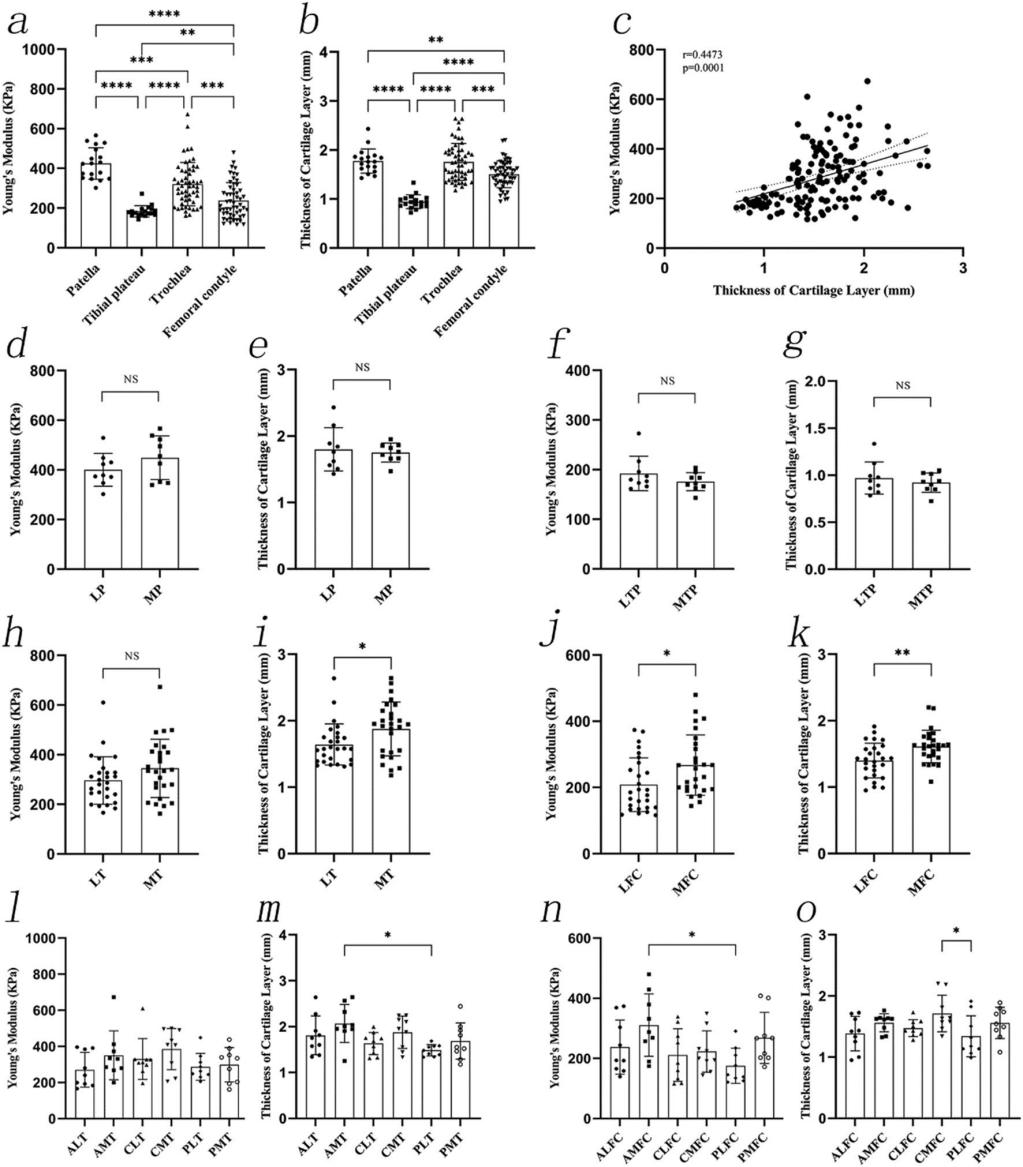
The Young's modulus and thickness of cartilage layer. **a**–**b**, The Young's modulus and thickness of cartilage layer from different sites. **c**, The correlation between the Young's modulus of cartilage and the thickness of the cartilage layer. **d**–**e**, The Young's modulus and thickness of cartilage at the LP and MP. **f**–**g**, The Young's modulus and thickness of cartilage at the LTP and MTP. **h**–**i**, The Young's modulus and thickness of cartilage at the LT and MT. **j**–**k**, The Young's modulus and thickness of cartilage at the LFC and MFC. **l**–**m** The Young's modulus and thickness of cartilage at six regions of the trochlea. **n**–**o**, The Young's modulus and thickness of cartilage at six regions of the femoral condyle. OCAs: osteochondral allografts; LP: lateral patella; MP: medial patella; LTP: lateral tibial plateau; MTP: medial tibial plateau. LT: lateral trochlea; MT: medial trochlea; LFC: lateral femoral condyle; MFC: medial femoral condyle; ALT: anterolateral trochlea; CLT: centrolateral trochlea; PLT: posterolateral trochlea; AMT: anteromedial trochlea; CMT: centromedial trochlea; PMT: posteromedial trochlea; ALFC: anterolateral femoral condyle; CLFC: centrolateral femoral condyle; PLFC: posterolateral femoral condyle; AMFC: anteromedial femoral condyle; CMFC: centromedial femoral condyle; PMFC: posteromedial femoral condyle

The results of the thickness of the cartilage layer showed that the patella cartilage layer was the thickest, followed by the trochlea, femoral condyle, and the tibial plateau (Table 2). The thickness of the medial and lateral cartilage layer was significantly different in the trochlea and femoral condyle where MT (1.88 ± 0.41 mm) was higher than the LT (1.64 ± 0.31 mm) and the MFC (1.61 ± 0.25 mm) was higher than the LFC (1.40 ± 0.26 mm). Subsequently, the analysis of cartilage thickness in the six regions of the trochlea and femoral condyle showed a relationship between AMT vs. PLT (2.07 ± 0.41 mm, 1.48 ± 0.12 mm, *P* = 0.0105), and CMFC vs. PLFC (1.71 ± 0.30 mm, 1.34 ± 0.34 mm, *P* = 0.0481), respectively. There was a moderate correlation between Young's modulus and the thickness of the cartilage layer (r = 0.4473, *P* < 0.0001) (Fig. 5).

**Table 2:** Mean articular cartilage thickness using different methods at different sites

Cartilage Source	Thickness (mm)		
	Histological Assessment (n = 4)	Nanoindentation measurements (n = 9)	unconfined compression test (n = 9)
Patella	1.89 ± 0.09	1.78 ± 0.25	1.72 ± 0.38
Tibial plateau	1.08 ± 0.18	0.95 ± 0.14	0.86 ± 0.19
Trochlea	1.75 ± 0.27	1.76 ± 0.38	1.71 ± 0.40
Femoral condyle	1.64 ± 0.38	1.51 ± 0.27	1.53 ± 0.32

### Uniaxial unconfined compression test

The compressive modulus of the different sites was significantly different. The compressive modulus of the patellar cartilage (2.11 ± 1.18 MPa) was the highest, followed by that of the trochlea (1.53 ± 0.85 MPa), femoral condyle (1.00 ± 0.66 MPa), and the tibial plateau (0.55 ± 0.41 MPa) (*P* < 0.0001). Furthermore, comparison of the compressive modulus of the lateral and medial AC showed that the compressive modulus in LP (1.77 ± 1.07 MPa), LTP (0.37 ± 0.26), LT (1.11 ± 0.63 MPa), and LFC (0.75 ± 0.51 MPa) was lower than in MP (2.46 ± 1.25 MPa, *P* = 0.2255), MTP (0.73 ± 0.47 MPa, *P* = 0.0595) MT (1.94 ± 0.84 MPa, P = 0.0002), and MFC (1.24 ± 0.70 MPa, *P* = 0.0055).

**Fig. 6:**
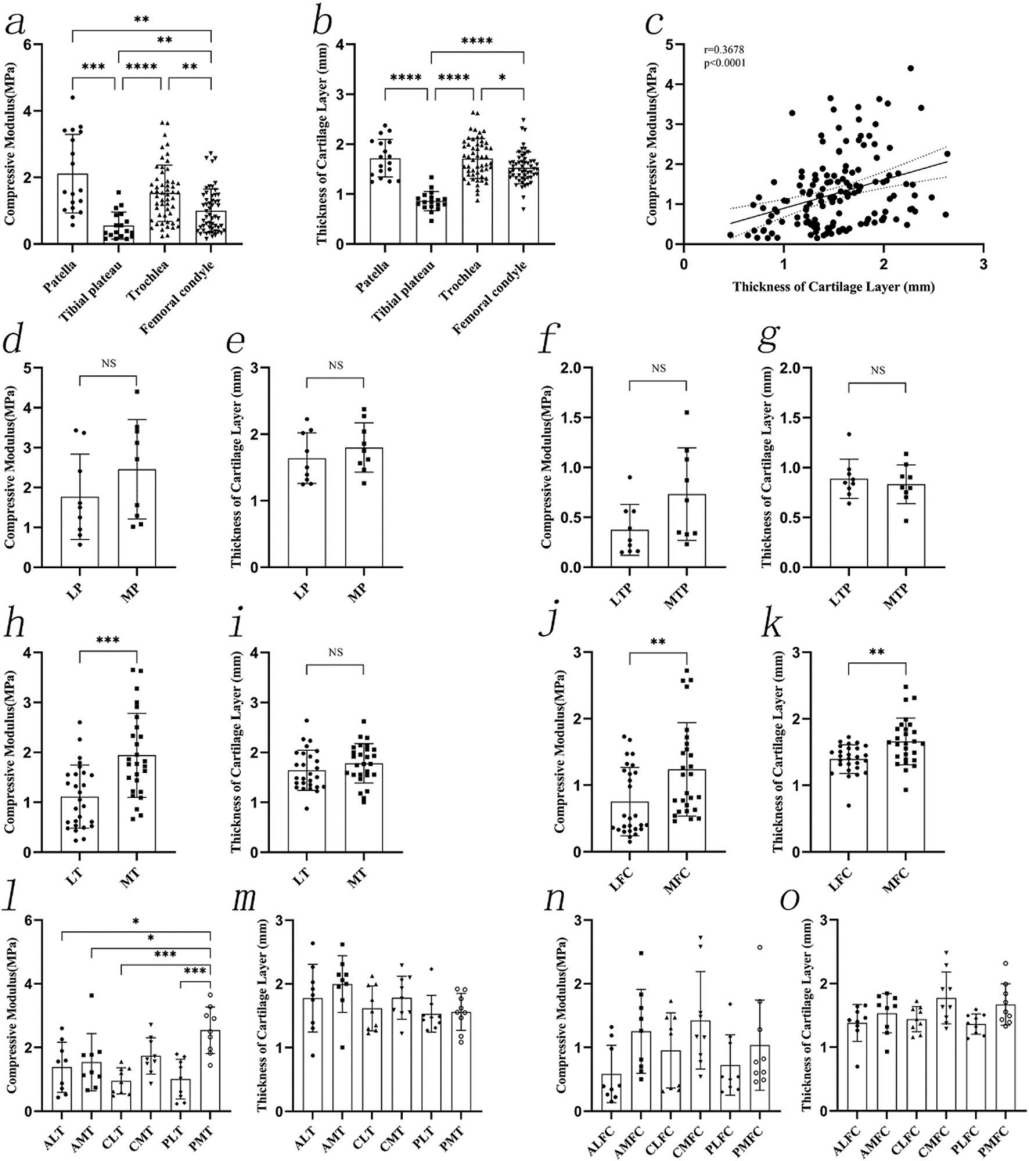
The compressive modulus and thickness of cartilage layer. **a**–**b**, The compressive modulus and thickness of cartilage layer in different sites. **c**, The correlation between the compressive modulus of cartilage and the thickness of the cartilage layer. **d**–**e**, The compressive modulus and thickness of cartilage at the LP and MP. **f**–**g**, The compressive modulus and thickness of cartilage at the LTP and MTP. **h**–**i**, The compressive modulus and thickness of cartilage at the LT and MT. **j**–**k**, The compressive modulus and thickness of cartilage at the LFC and MFC. **l**–**m**, The compressive modulus and thickness of cartilage at six regions of the trochlea. **n**–**o**, The compressive modulus and thickness of cartilage at six regions of the femoral condyle. OCAs: osteochondral allografts; LP: lateral patella; MP: medial patella; LTP: lateral tibial plateau; MTP: medial tibial plateau. LT: lateral trochlea; MT: medial trochlea; LFC: lateral femoral condyle; MFC: medial femoral condyle; ALT: anterolateral trochlea; CLT: centrolateral trochlea; PLT: posterolateral trochlea; AMT: anteromedial trochlea; CMT: centromedial trochlea; PMT: posteromedial trochlea; ALFC: anterolateral femoral condyle; CLFC: centrolateral femoral condyle; PLFC: posterolateral femoral condyle; AMFC: anteromedial femoral condyle; CMFC: centromedial femoral condyle; PMFC: posteromedial femoral condyle

Subsequent analysis of the compressive modulus at the six regions of the trochlea showed the following relationships: ALT vs. PMT (*P* = 0.0120), AMT vs. PMT (*P* = 0.0499), CLT vs. PMT (*P* = 0.0002), and PLT vs. PMT (*P* = 0.0003). The trochlea regions with the highest and lowest compressive modulus were the PMT (2.54 ± 0.73 MPa) and CLT (0.95 ± 0.41 MPa), respectively. Other post-hoc test results were not statistically significant. There were no statistically significant differences between the compressive modulus in the six regions of the femoral condyle. The regions of the femoral condyle with the highest and lowest compressive modulus were the CMFC (1.43 ± 0.76 MPa) and ALFC (0.58 ± 0.45 MPa), respectively. The results of the cartilage layer thickness showed that the patella cartilage layer was the thickest, followed by the trochlea, the femoral condyle, and the tibial plateau (Table 2). The thickness of the medial and lateral cartilage layer had significant differences in the femoral condyle such that the thickness of MFC (1.66 ± 0.35 mm) was higher than that of LFC (1.40 ± 0.22 mm). The regions of the trochlea with the highest and lowest thickness were AMT (2.00 ± 0.45 mm) and PLT (1.53 ± 0.29 mm), respectively. The regions of the femoral condyle with the highest and lowest thickness were CMFC (1.77 ± 0.41 mm) and PLFC (1.36 ± 0.16 mm), respectively. The compressive modulus and the thickness of the cartilage layer showed a significant correlation (r = 0.3678, *P* < 0.0001) (Fig. 6).

### Histological assessment

**Fig. 7:**
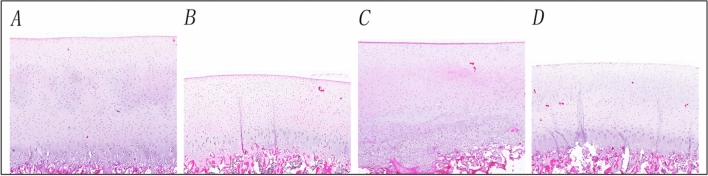
HE staining results of different sites. **A**–**D**, The staining results of OCAs in the patella, tibial plateau, trochlea, femoral condyle, respectively. OCAs: osteochondral allografts

The cartilage thicknesses of the different sites were different (Fig. 7). Histological assessment of indicated the patellar cartilage (1.89 ± 0.09 mm) was the thickest, followed by the trochlea (1.75 ± 0.27 mm), femoral condyle (1.64 ± 0.38 mm), and tibial plateau (1.08 ± 0.18 mm) (Table 2).

## Discussion

The cartilage at different sites is exposed to different magnitudes of forces in the knee joint. (Li et al. 2021a, Mieloch et al. 2019) The biomechanical properties of AC have been studied extensively, but most studies have concentrated on the overall biomechanical properties of cartilage without considering specialized regional variations. (McCready et al. 2022, Risch et al. 2021) Only a few studies have described the biomechanical properties of the medial and lateral AC at different sites. (Li et al. 2021a, Risch et al. 2021) Our study aimed to characterize the biomechanical properties and compare the thickness of AC in different regions and different sites of knee joints. These results highlight the importance of investigating different subregions at different sites and offer a novel method for OCA transplantation to ensure the consistency of biomechanical properties and cartilage thickness of the donor and recipient.

Osteochondral transplantation (OT) involves the transplantation of mature, active hyaline cartilage and supporting subchondral bone into the area of the cartilage defect. (Krych et al. 2020) OT is a restorative cartilage technique that can improve joint function in patients with symptomatic articular cartilage (AC) defects in various joints, including the knee, hip, ankle, and shoulder. Currently, it is commonly used to treat knee cartilage injuries. (Familiari et al. 2018, Luk et al. 2021, Stannard and Cook 2020) OT includes osteochondral autograft transfer (OAT) and OCA. OAT entails harvesting and transferring an osteochondral plug from a non-weight bearing area of the knee, usually the trochlea, to a weight-bearing chondral lesion. (Krych et al. 2020) A limitation of this approach is the challenge in matching the contour of the donor cartilage with that of the lesion to achieve a congruent surface recovery. (Krych et al. 2020) Significant differences have been observed in the biomechanical properties and thickness of the trochlea and femoral condyle cartilage, which may be a cause of OAT failure. OCA transplantation was successful for all types of osteochondral injuries. (Gilat et al. 2021, Krych et al. 2020, Stannard and Cook 2020) AC injuries at various anatomic sites of the knee, such as the patella, (Melugin et al. 2021, Mirzayan et al. 2020) tibial plateau, (Liu et al. 2020) trochlea, (Mirzayan et al. 2020) and femoral condyle, (Gilat et al. 2021, Gómez Cimiano et al. 2021, Tirico et al. 2019) can be treated using OCA transplantation with good outcomes.

The results of our study showed that the patellar cartilage was the stiffest, followed by the trochlea, femoral condyle, and tibial plateau. These results were similar to studies performed on canine knee cartilage. (Jurvelin et al. 2000, Li et al. 2021a) Jurvelin et al. (2000) observed different variations of the biomechanical properties of cartilage in knee joints, and showed that the stiffest and softest cartilage were the trochlea and tibial plateau, respectively. In addition, Li et al. (2021a) reported the topographic variation of the elastic properties of AC and showed that the stiffest and softest cartilage were the trochlea and MTP, respectively. However, they did not study the patellar cartilage and believed that the trochlea was the stiffest. Our study also observed biomechanical differences within the same site, especially in the trochlea and femoral condyle. The biomechanics of AC in MT and MFC were significantly higher than in LT and LFC, respectively, which indicates the necessity of investigating different subregions at different sites. Therefore, we further divided the trochlea and femoral condyle into six regions each. The main difference after AC regionalization was found between the inside and outside compartments; however, subregion transplantation may be more conducive to donor-host matching from biomechanical and morphological perspectives. Many studies have shown the importance of matching the AC surface of the knee joint with the natural topographic anatomy during OCA transplantation. (Bernstein, et al. 2017, Gursoy et al. 2021, Kock et al. 2008) The failure to match these components may negatively affect clinical outcomes (Gursoy et al. 2021, Nakagawa, et al. 2007) and subregional transplantation may be the solution. There was no significant difference in biomechanical properties between LP and MP, or between LTP and MTP; therefore, no more detailed division was required.

Under physiological loads, the biomechanical properties of cartilage tissue depend on the functional demand of the joints. (Li et al. 2021a) AC showed a distinct regional difference, probably related to physiological function. This includes the transfer of load between the joint surfaces and the movement of the knee joint. The results of the nano-indentation measurement and the unconfined compression test indicated that the cartilage of the tibial plateau and femoral condyle were softer and the patella and trochlea had stronger biomechanical properties. These could reflect the difference in physiological demand in the knee joint. The stiffer sites are the patella and trochlea, probably because they are the areas of contact with the joints. (Li et al. 2021a, Thambyah et al. 2006) In addition, the tibial plateau and the femoral condyle are covered by a meniscus that transmits powers to the cartilage, absorbs load, and dissipates pressure, resulting in soft but thin AC (Li et al. 2021a).

The availability of OCAs is currently limited due to several constraints. To ensure suitable graft size and chondral surface geometry, precise recipient–donor size matching and laterality matching are necessary. (Babu, et al. 2020, Bernstein et al. 2017) Our observation found that 7 mm OCA presented superior surface biomechanical properties than 4 mm OCA and cartilage grafts. Babu et al. (2020) studied the capacity of the difference OCA depth to resist displacement and found that 7 mm OCAs displayed significantly better resistance to pull out than 4 mm OCAs and comparable resistance to subside and pull out as 10 mm OCAs. The general recommendation for clinicians performing OCA transplantation is to minimize the bone portion of the OCAs. (Elder et al. 2018, Luk et al. 2021, Meyer et al. 2017, Neunaber et al. 2022) The reason is that longer grafts can produce immune rejection, resulting in slow resorption and incomplete healing. (van Dijk 2017) Studies have shown that the bone portion of OCAs must be minimized so that the maximum is 5–6 mm to benefit effective integration of the recipient–donor. (Luk et al. 2021) During OCA transplantation, an OCA of 7 mm in depth is the ideal length.

The AC thickness is a commonly reported outcome, and measurements are commonly performed using computed tomography (CT), magnetic resonance imaging (MRI), vernier calipers, or histologically. (Appleyard et al. 2003, Horbert et al. 2019, Malda et al. 2012, 2013, Pflieger et al. 2019, Risch et al. 2021) We chose Vernier caliper and histology approaches to measure the thickness of AC to reduce the error caused by differences in measurement methods, and the result showed that there was no significant difference between the two methods. Sidharthan et al. (2021) studied the thickness of AC in pediatric and adolescent knees using MRI and showed that AC was thickest in the patella, followed by the trochlea, the femoral condyle, and the tibial plateaus. Furthermore, Sidharthan et al. (2021) showed that the mean cartilage thickness differed significantly between various anatomic sites; however, post hoc tests revealed that there was no significant difference in cartilage thickness between LP and MP or LFC and LTP. These results show that the cartilage of pigs is similar to that of human cartilage.

This study had some limitations. The health of the AC surface was assessed macroscopically; however, some subtle degenerations could not be visually detected. However, the same common trend could be observed in all samples, which was similar to other studies. (Li et al. 2021a, Sidharthan et al. 2021) Furthermore, osteochondral samples were meticulously designed to achieve a flat surface; however, the natural curvatures of the native cartilage surface may have led to subtle inaccuracies in the determination of the contact point during compressive testing, which would result in minor errors in modulus calculations. Finally, the subregions of AC were evaluated based on biomechanics and thickness. Biochemical analysis was not performed to elucidate the internal structure and composition of the cartilage.

In summary, this study demonstrated that 7-mm OCAs exhibit a larger Young's modulus compared to 4-mm OCAs and cartilage layer grafts, indicating that 7-mm OCAs may be more suitable for transplantation from a biomechanical perspective. Furthermore, the AC in different regions of the knee joint displays distinct biomechanical properties and thickness, and shows a correlation between biomechanics and AC thickness. Therefore, during OCA transplantation, it is advisable to transplant OCAs in the same or nearest regions possible to maintain consistent biomechanical properties and cartilage thickness between the donor and recipient, ensuring smooth integration with the surrounding tissue.

## Data Availability

The data that support the findings of this study are available from the corresponding author, [D], upon reasonable request.
